# The Calcium-Activated Chloride Channel TMEM16A is Inhibitied by Liquiritigenin

**DOI:** 10.3389/fphar.2021.628968

**Published:** 2021-04-08

**Authors:** Mami Kato, Yasunori Takayama, Masataka Sunagawa

**Affiliations:** Department of Physiology, Showa University School of Medicine, Tokyo, Japan

**Keywords:** TMEM16A, TRP channel, flavonoid, Liquiritigenin, Estrogen, aglycone

## Abstract

The transmembrane 16 (TMEM16) family contains 10 subtypes, and the function of each protein is different. TMEM16A is a calcium-activated chloride channel involved in physiological and pathological situations. Liquiritigenin is an aglycone derived from *Glycyrrhiza glabra*, and it is generated via the metabolism of enterobacterial flora. It has been known that liquiritigenin reduces pain sensation involving TMEM16A activation in primary sensory neurons. In addition, other pharmacological effects of liquiritigenin in physiological functions involving TMEM16A have been reported. However, the relationship between TMEM16A and liquiritigenin is still unknown. Therefore, we hypothesized that TMEM16A is inhibited by liquiritigenin. To confirm this hypothesis, we investigated the effect of liquiritigenin on TMEM16A currents evoked by intracellular free calcium in HEK293T cells transfected with TMEM16A. In this study, we found that liquiritigenin inhibited the mouse and human TMEM16A currents. To further confirm its selectivity, we also investigated its pharmacological effects on other ion channels, including transient receptor potential vanilloid 1 (TRPV1) and ankyrin 1 (TRPA1), which are non-selective cation channels involved in pain sensation. However, liquiritigenin did not inhibit the currents of TRPV1 and TRPA1 induced by capsaicin and allyl isothiocyanate, respectively. Therefore, our findings indicate that selective TMEM16A inhibition could be one molecular mechanism that explains liquiritigenin-induced pain reduction. Additionally, we also investigated the inhibitory effects of estrogens on TMEM16A because liquiritigenin reportedly binds to the estrogen receptor. In this study, a pregnancy-dependent estrogen, estriol, significantly inhibited TMEM16A. However, the efficacy was weak. Although there is a possibility that TMEM16A activity could be suppressed during pregnancy, the physiological significance seems to be small. Thus, the inhibitory effect of estrogen might not be significant under physiological conditions. Furthermore, we investigated the effect of dihydrodaidzein, which is an analog of liquiritigenin that has a hydroxyphenyl at different carbon atom of pyranose. Dihydrodaidzein also inhibited mouse and human TMEM16A. However, the inhibitory effects were weaker than those of liquiritigenin. This suggests that the efficacy of TMEM16A antagonists depends on the hydroxyl group positions. Our finding of liquiritigenin-dependent TMEM16A inhibition could connect the current fragmented knowledge of the physiological and pathological mechanisms involving TMEM16A and liquiritigenin.

## Introduction

TMEM16A (also called anoctamin 1, ANO1) belongs to the TMEM16 family and is a calcium-activated chloride channel (Caputo et al., 2008; Schroeder et al., 2008; Yang et al., 2008). This ion channel is expressed in primary sensory neurons and many epithelial cells (Yang et al., 2008) and plays important roles in various physical functions, including nociception (Cho et al., 2012), intestinal peristalsis (Huang et al., 2009; Zhu et al., 2009), mucin secretion (Namkung et al., 2011; Huang et al., 2012), and insulin release (Xu et al., 2014; Crutzen et al., 2016). TMEM16A can be activated by noxious heat in dorsal root ganglia neurons and causes burning pain (Cho et al., 2012). Furthermore, TMEM16A is also involved in inflammatory pain induced by bradykinin (Lee et al., 2014).

In addition to TMEM16A, several ion channels responsible for noxious stimuli are expressed in primary sensory neurons. In particular, transient receptor potential vanilloid 1 (TRPV1) and TRP ankyrin 1 (TRPA1) are known to be activated by various nociceptive stimuli and natural compounds. TRPV1 is activated by capsaicin, resiniferatoxin, piperine, camphor, noxious heat, acidic pH, and double-knot toxin in tarantula venom (Julius, 2013), and TRPA1 is activated by allyl isothiocyanate, allicin, cinnamaldehyde, oxidative stress, and cold stimuli (Gao et al., 2020). The interaction of ion channels associated with TRPV1 is important for modulation of nociceptor activity (Weng et al., 2015; Patil et al., 2020). A recent study also suggested that local calcium influx through TRPV1 and calcium release from the endoplasmic reticulum activated TMEM16A, and this activation was followed by pain enhancement through additional depolarization resulting from TRPV1/TMEM16A interactions (Shah et al., 2020). Blocking TMEM16A activity with TMEM16A inhibitors reduces TRPV1-mediated pain-related behaviors in mice (Oh and Jung, 2016). Moreover, emerging evidence has suggested that pharmacological inhibition of TMEM16A might be beneficial for the treatment of TMEM16A-associated diseases such as asthma (Huang et al., 2012), vasoconstriction of cerebral arteries (Wang et al., 2016), and diarrhea (Ko et al., 2014). These previous studies indicate that the development of a TMEM16A inhibitor could lead to promising new treatments to reduce physical symptoms in certain medical conditions.

Some natural compounds are candidate TMEM16A inhibitors, such as flavonoids (Seo et al., 2017; Zhang et al., 2017). In this study, we performed whole-cell patch-clamp recordings and identified that a licorice-derived flavonoid, liquiritigenin, inhibited TMEM16A currents in HEK293T cells. Liquiritigenin is an aglycone that is metabolized from a glycoside of licorice, *Glycyrrhizae radix*, by enterobacteria. This flavonoid selectively activates an estrogen receptor β (Mersereau et al., 2008; Powell and Xu, 2008; Jiang et al., 2013). Liquiritigenin is maintained in the plasma over 24 h after oral administration of *Glycyrrhizae radix* extract (Han et al., 2019). Many pharmacological effects of liquiritigenin have been reported, such as anti-inflammatory effects, reduction of pain sensation, neuroprotection, and anti-cancer effects (Ramalingam et al., 2018). Furthermore, the influenza A virus could be suppressed by TMEM16A inhibitors (Pearson et al., 2020), and an anti-influenza effect of liquiritigenin has been reported (Grienke et al., 2014). There are no reports investigating the liquiritigenin effects on TMEM16A, although liquiritigenin has been reported to inhibit ion channels, including the voltage-gated sodium channel subtype 1.4 (Zhu et al., 2018), TRPM3, which is involved in noxious thermal pain (Straub et al., 2013; Vandewauw et al., 2018), and 5-HT3A receptors (Herbrechter et al., 2015).

## Materials and Methods

### Chemicals

Liquiritin, liquiritigenin, estrone (E1), 17β-estradiol (E2), and estriol (E3) were purchased from FUJIFILM Wako (Japan). Capsaicin, estetrol (E4), and Ani9 were purchased from Sigma-Aldrich (United States). Allyl isothiocyanate, niflumic acid, and dihydrodaidzein were purchased from Tokyo Chemical Industry (Japan).

### Cells

HEK293T cells were cultured in Dulbecco’s Modified Eagle Medium (high glucose) with l-glutamine and phenol red (FUJIFILM Wako, Japan), containing 10% fetal bovine serum (lot# G121–6, JR Scientific, United States), penicillin/streptomycin (1:100, FUJIFILM Wako, Japan), and Glutamax (1:100, Thermo Fisher Scientific, United States), at 37°C in a humidified chamber containing 5% CO_2_.

### cDNA Plasmids

Mouse *Tmem16a* and *Tmem16b* plasmids were a generous gift from Dr Uhtaek Oh (Korea Institute of Science and Technology). Human *TMEM16A* untagged complete CDS plasmid was purchased from OriGene (United States). Mouse and human *TRPA1* plasmids were a generous gift from Dr Ardem Patapoutian (Howard Hughes Medical Institute). Mouse *Trpv1* plasmid was a generous gift from Dr Michael Zhu (University of Texas). Human *TRPV1* plasmid was a generous gift from Dr Yasuo Mori (Kyoto University). Each plasmid was amplified in XL1 Blue supercompetent cells (Agilent Technologies, United States) and purified using NucleoBond PC 500 EF (Macherey-Nagel, Germany). All experiments using plasmid vectors were approved by the Biosafety Committee of Showa University School of Medicine (approved number: 2010).

### Whole-Cell Patch-Clamp Recording

The cells were transfected with 0.5 µg of cDNA plasmid using Lipofectamine 3,000 (Invitrogen, United States) and were used from 20 to 30 h after transfection. The bath solution contained 140 mM NaCl, 5 mM KCl, 2 mM MgCl_2_, 5 mM ethylene glycol tetraacetic acid (EGTA) or 2 mM CaCl_2_ (for TRP channels or TMEM16A, respectively), 10 mM d-glucose, and 10 mM HEPES, pH 7.4, adjusted with NaOH. The pipette solution contained 140 mM NMDG-Cl or CsCl, 5 mM 1.2-bis(*o*-aminophenoxy)ethane-N,N,N′,N′-tetraacetic acid (BAPTA), and 10 mM HEPES, pH 7.4, adjusted with NMDG or CsOH. CsCl-contained pipette solution was used in the investigation of agonistic effects of liquiritigenin on TRP channels. The free calcium concentration in the pipette solution was calculated using the MAXC program (Stanford University). The intracellular free calcium concentration that activates mTMEM16A and hTMEM16A in transfected HEK293T cells was determined before the experiment (Supplementary Figure S1). Pipette resistances were 4 ± 1 MΩ. The holding potential was −60 mV or 0 mV, and ramp pulses from −100 mV to +100 mV were applied for 300 ms every 5 s. Currents were recorded using a Multiclamp 700 B amplifier (Molecular Devices, United States), filtered at 1 kHz with a low-pass filter, and digitized with a Digidata 1550 B digitizer (Molecular Devices, United States). Data were acquired with pCLAMP 11 (Axon Instruments, United States).

### Statistical Analyses

Statistical analyses were performed with Origin Pro 2020b (OriginLab, United States). Wilcoxon matched-pairs signed rank test and Kruskal-wallis ANOVA were performed for comparisons between groups. A value of *p* < 0.05 indicated a statistically significant difference.

## Results

### Inhibitory Effect of Liquiritigenin on Mouse TMEM16A

**FIGURE 1:**
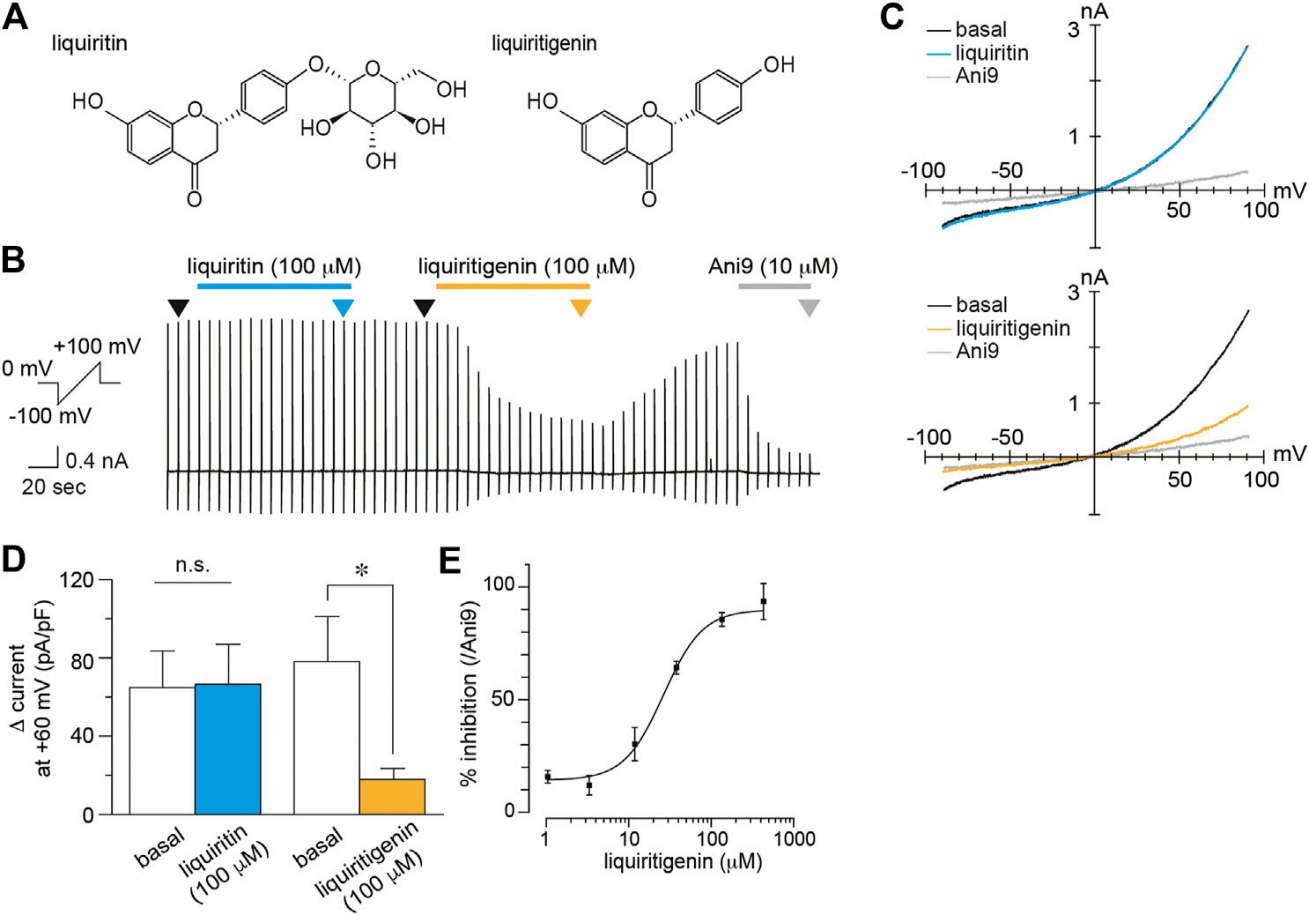
Inhibition of mTMEM16A currents by liquiritigenin **(A)** Chemical structure of liquiritin **(left)** and liquiritigenin **(right)** **(B**–**C)** A typical trace **(B)** and current-voltage curves **(C)** of whole-cell chloride currents during application of 100 µM liquiritin and 100 µM liquiritigenin to HEK293T cells expressing mTMEM16A. The holding potential was 0 mV, and ramp pulses (−100 to +100 mV, 300 ms) were applied every 5 s. Basal TMEM16A currents were induced by 100 nM intracellular free calcium. Current-voltage curves at the time indicated by black (basal), cyan (liquiritin), yellow (liquiritigenin), and gray (Ani9) arrowheads in **(B)**. **(D)** Comparison of the Δ current at +60 mV (liquiritin; n = 7 cells, liquiritigenin; n = 8 cells). Data are shown as the mean ± SEM; *p < 0.05, n. s., not significant, Wilcoxon matched-pairs signed rank test **(E)** Dose-response curve for liquiritigenin-induced inhibition of mTMEM16A currents at +60 mV (n = 5–6 cells). The IC_50_ was 21.32 µM.

**FIGURE 2:**
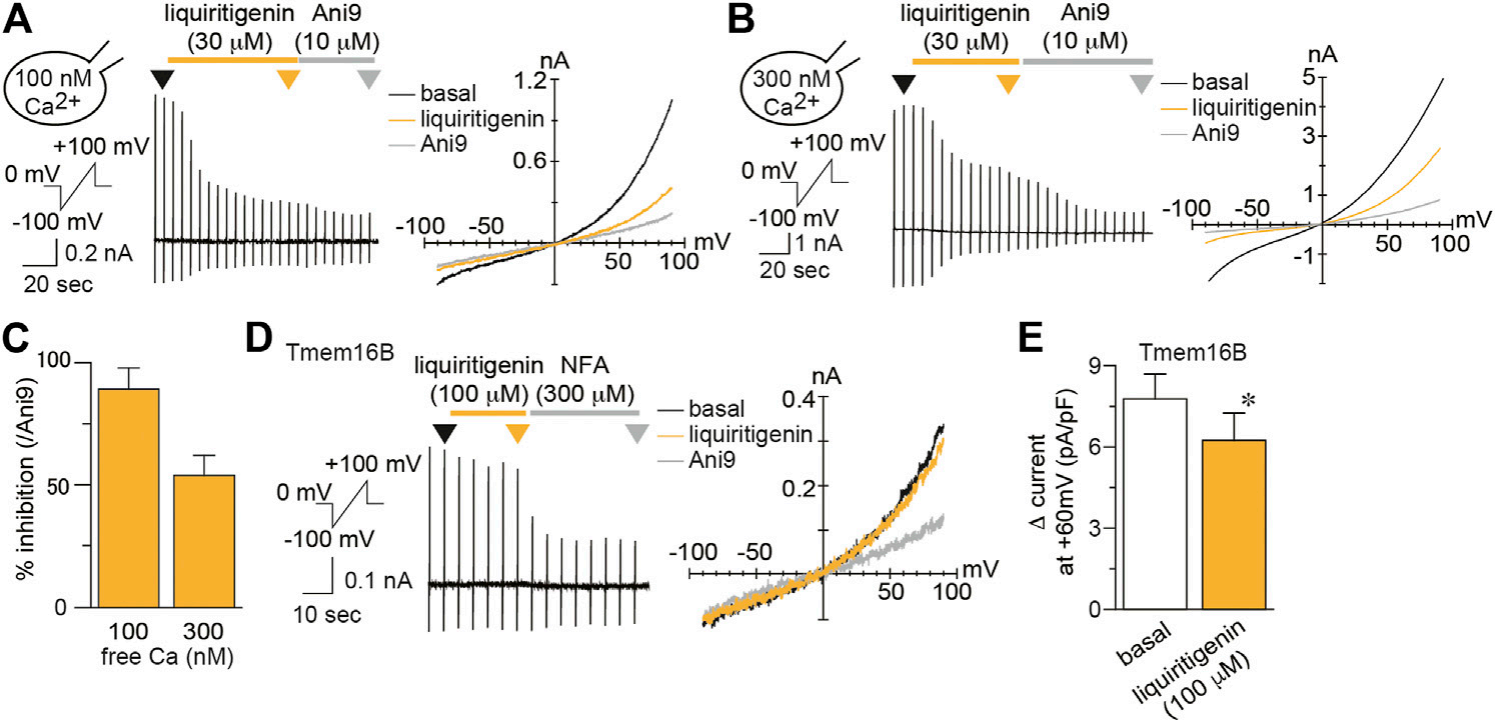
Characterization of liquiritigenin **(A**–**B)** Typical traces **(left)** and current-voltage curves **(right)** of mTMEM16A currents induced by 100 nM **(A)** or 300 nM **(B)** intracellular free calcium during application of 30 µM liquiritigenin to HEK293T cells expressing mTMEM16A. The holding potential was 0 mV, and ramp pulses (−100 to +100 mV, 300 ms) were applied every 5 s. Typical current-voltage curves at the time indicated by black (basal), yellow (liquiritigenin), and gray (Ani9) arrowheads **(C)** The percentage of inhibition caused by 30 µM liquiritigenin applied with 100 and 300 nM intracellular free calcium concentrations (n = 5–6 cells) **(D)** A typical trace **(left)** and current-voltage curves **(right)** of whole-cell chloride currents during application of 100 µM liquiritigenin in HEK293T cells expressing mTMEM16B. The holding potential was 0 mV, and ramp pulses (−100 to +100 mV, 300 ms) were applied every 5 s. Basal mTMEM16B currents were induced by 300 nM intracellular free calcium. Typical current-voltage curves at the time indicated by black (basal), yellow (liquiritigenin), and gray (niflumic acid, NFA) arrowheads **(E)** Comparison of the Δ currents in HEK293T cells expressing mTMEM16B at +60 mV (n = 5 cells). Data are shown as the mean ± SEM; *p < 0.05, Wilcoxon matched-pairs signed rank test.

To identify the effects of liquiritigenin on TMEM16A currents, we performed whole-cell patch-clamp recording in HEK293 T cells expressing mouse TMEM16A (mTMEM16A). mTMEM16A currents were induced by the intracellular free calcium concentration (100 nM). In this exploratory study, we found that TMEM16A currents were inhibited by liquiritigenin (Figure 1). The current recovery rate after washing liquiritigenin out seemed to be unstable, although the inhibitory effect was reversible (Supplementary Figure S2). The analyzed values (Δ current) were obtained by subtracting the peak current value after the application of a selective TMEM16A inhibitor, 10 μM Ani9 (Figure 1D). The current density was significantly decreased after liquiritigenin application (Wilcoxon matched-pairs signed rank test, n = 8). Additionally, we investigated the effects of liquiritin, which is a glycoside of liquiritigenin; however, it had no effect on mTMEM16A activity (Wilcoxon matched-pairs signed rank test, n = 7). Figure 1E shows the normalized dose-response curve of liquiritigenin (% inhibition). These values were calculated as the ratio to the maximum reduction by Ani9 (10 µM) administration. Liquiritigenin exhibited concentration-dependent inhibition, and the half-maximal inhibitory concentration (IC_50_) was 21.32 μM at a membrane potential of +60 mV (n = 5–6). TMEM16A activity is enhanced by increases in the intracellular free calcium concentration (Caputo et al., 2008; Yang et al., 2008). To establish the inhibitory effect under the highly active condition of TMEM16A, we compared TMEM16A currents induced by low and high free calcium concentrations in the pipette solutions (Figures 2A–C). In these experiments, the % inhibition values induced by liquiritigenin (30 µM) with 100 and 300 nM free calcium concentrations were 88.22 and 53.42%, respectively (n = 5–6). These results show that liquiritigenin inhibits mTMEM16A, although the effect is inversely proportional to the strength of its activation.

To investigate its selectivity, we examined the effect of liquiritigenin on mouse TMEM16B (mTMEM16B, also called anoctamin 2, ANO2), another TMEM16 subtype (Figures 2D,E). TMEM16B is also a calcium-activated chloride channel, although its calcium sensitivity is lower than that of TMEM16A (Schroeder et al., 2008). mTMEM16B currents were induced by high intracellular free calcium (300 nM), and mTMEM16B was weakly activated in our experiments. We calculated the Δ current compared with niflumic acid (300 µM), a non-selective chloride channel inhibitor (Pifferi et al., 2009). Under this condition, the inhibitory effect of liquiritigenin (100 µM) was quite weak, although a significant difference was found (Wilcoxon matched-pairs signed rank test, n = 5, Figures 2D,E). Therefore, it is reasonable to assume that liquiritigenin selectively inhibits mTMEM16A.

### Liquiritigenin Does Not Inhibit Mouse TRPV1 and TRPA1

Certain receptor-type ion channels are affected by environmental factors. In particular, TMEM16A expressed in primary sensory neurons is involved in nociception together with TRP channels (Pedemonte and Galietta, 2014). Therefore, we analyzed the inhibitory effects of liquiritigenin on the currents of major TRP channels, including mouse TRPV1 and TRPA1, expressed in nociceptors (Supplementary Figures S3A,E). We maintained the basal membrane potential at 0 mV during TRPV1 current recordings because TRPV1-dependent ion dynamics can change the cell size depending on whether the membrane potential is positive or negative; for instance, the cell is expanded at negative potentials during TRPV1 activation. Liquiritigenin (100 µM) did not inhibit mTRPV1 and mTRPA1 currents induced by capsaicin (100 nM) and allyl isothiocyanate (300 µM), respectively (Kruskal-wallis ANOVA, n = 10, Supplementary Figures S3B, F). These results suggest that liquiritigenin has selectivity for ion channels, although it reportedly inhibits voltage-gated sodium channels, TRPM3, and 5-HT receptors (Straub et al., 2013; Herbrechter et al., 2015; Zhu et al., 2018).

### Liquiritigenin has Similar Effects on Human TMEM16A, TRPV1, and TRPA1

**FIGURE 3:**
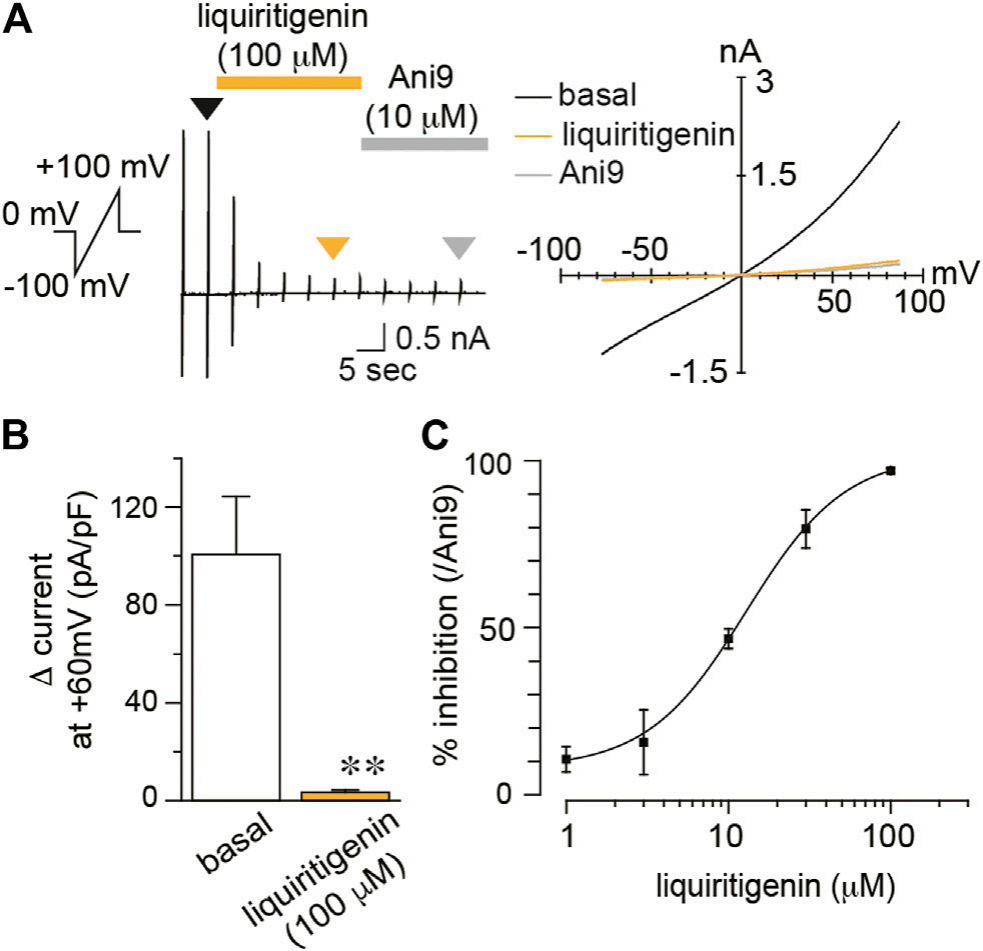
The inhibitory effects of liquiritigenin on human TMEM16A **(A)** A typical trace and current-voltage curves of chloride currents during the application of 100 µM liquiritigenin to HEK293T cells expressing hTMEM16A. Typical current-voltage curves at the time indicated by black (basal), yellow (liquiritigenin), and gray (Ani9) arrowheads **(B)** Comparison of the Δ current in HEK293T cells expressing hTMEM16A at +60 mV (n = 6 cells). Data are shown as the mean ± SEM; **p < 0.01, Wilcoxon matched-pairs signed rank test **(C)** A dose-response curve for liquiritigenin-induced inhibition of hTMEM16A currents at +60 mV (n = 5–6 cells). The IC_50_ was 12.89 µM.

The agonistic or antagonistic effects of natural compounds on ion channels differ depending on the animal species. For instance, caffeine activates and inhibits TRPA1 in mouse and human, respectively (Nagatomo and Kubo, 2008). To examine the different effects of liquiritigenin between species, we also analyzed human TMEM16A (hTMEM16A), TRPV1 (hTRPV1), and TRPA1 (hTRPA1) currents. hTMEM16A currents induced by intracellular free calcium concentration (300 nM) were almost completely inhibited by liquiritigenin (100 μM, Wilcoxon matched-pairs signed rank test, n = 6, Figure 3). The IC_50_ was 12.89 μM at a membrane potential of +60 mV (Figure 3C). The reversible currents after washing liquiritigenin out were similar in mTMEM16A (Supplementary Figure S4). These results indicate that the inhibitory effect of liquiritigenin on TMEM16A is stronger in human than in mouse channels.

Next, we examined the effects of liquiritigenin on hTRPV1 and hTRPA1. Similar to mouse clones, neither hTRPV1 (Supplementary Figures S3C,D) nor hTRPA1 (Supplementary Figures S3G,H) currents were inhibited by liquiritigenin (100 μM, Kruskal-wallis ANOVA, n = 10, Supplementary Figures S3D,H). These results demonstrate the selective effects of liquiritigenin on ion channel activity.

### Liquiritigenin Does Not Have Agonistic Effects on TRPV1 and TRPA1

To investigate the agonistic effects of liquiritigenin on TRPV1 and TRPA1, we performed whole-cell patch-clamp recordings in HEK293 T cells expressing both mouse and human TRPV1 or TRPA1. In these experiments, CsCl was used in the pipette solution to eliminate the endogenous voltage-dependent potassium currents in HEK293T cells and to clarify the outward currents of TRPV1 and TRPA1 because these TRP channels are permeable to cesium. We applied liquiritigenin (100 µM) to HEK293T cells expressing mTRPV1 or hTRPV1. In this experiment, no currents were induced by liquiritigenin, although TRPV1 currents were evoked by application of 1 µM capsaicin (n = 10, Wilcoxon matched-pairs signed rank test, Supplementary Figures S5A–D). Furthermore, mTRPA1 and hTRPA1 currents were not induced by liquiritigenin (100 µM), although allyl isothiocyanate (300 µM)-induced currents were observed (n = 10, Wilcoxon matched-pairs signed rank test, Supplementary Figures S5E–H). These results reveal that liquiritigenin has no effect on either mouse or human TRPV1 and TRPA1.

### Estrogen Slightly Inhibits TMEM16A

**FIGURE 4:**
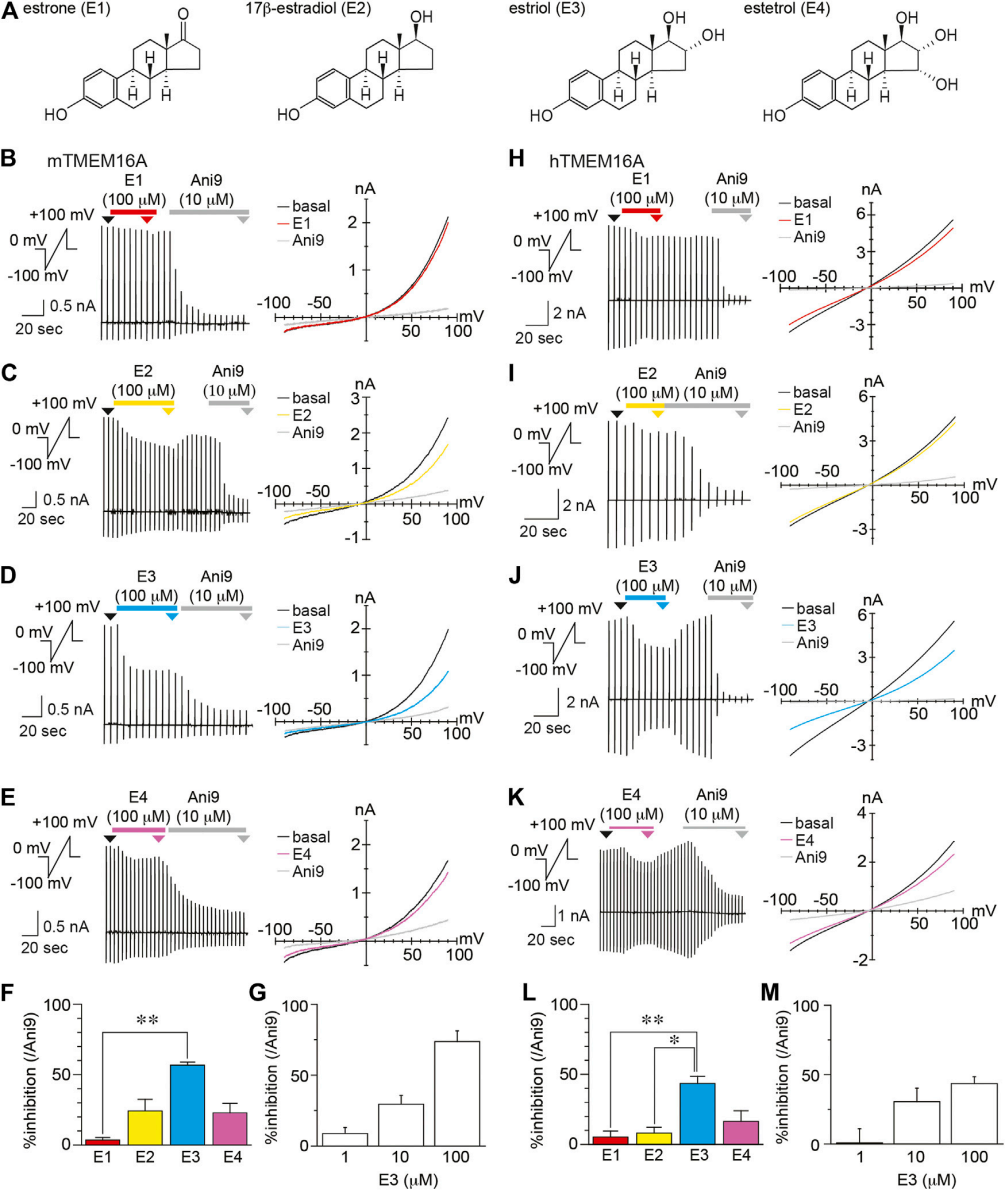
The inhibitory effects of estrogen on TMEM16A **(A)** Chemical structures of estrogen **(B**–**E)** Typical traces **(left)** and current-voltage curves **(right)** of chloride currents in HEK293T cells expressing mTMEM16A during application of estrone (E1) **(B)**, 17β-estradiol (E2) **(C)**, estriol (E3) **(D),** and estetrol (E4) **(E)**. The holding potential was 0 mV, and ramp pulses (−100 to +100 mV, 300 ms) were applied every 5 s. Basal mTMEM16A currents were induced by 100 nM intracellular free calcium. Typical current-voltage curves at the time indicated by black (basal), red (E1), yellow (E2), cyan (E3), violet (E4), and gray (Ani9) arrowheads **(F)** The percentage of inhibition caused by 100 µM estrogen at +60 mV (n = 5–6 cells) **(G)** The percentage of inhibition caused by 1, 10, and 100 µM E3 at +60 mV (n = 5 cells). Data are shown as mean ± SEM. **p < 0.01, Kruskal-wallis ANOVA **(H–K)** Typical traces **(left)** and current-voltage curves **(right)** of chloride currents in HEK293T cells expressing hTMEM16A during application of E1 **(H)**, E2 **(I)**, E3 **(J),** and E4 **(K)**. The holding potential was 0 mV, and ramp pulses (−100 to +100 mV, 300 ms) were applied every 5 s. Basal hTMEM16A currents were induced with 300 nM intracellular free calcium. Typical current-voltage curves at the time indicated by black (basal), red (E1), yellow (E2), cyan (E3), violet (E4), and gray (Ani9) arrowheads **(L)** The percentage of inhibition caused by 100 µM estrogen at +60 mV (n = 5 cells) **(M)** The percentage of inhibition caused by 1, 10, and 100 µM E3 at +60 mV (n = 3–5 cells). Data are shown as the mean ± SEM; **p < 0.01, *p < 0.05, Kruskal-wallis ANOVA.

According to previous reports, liquiritigenin is a high-affinity agonist of estrogen receptor β (Mersereau et al., 2008; Powell and Xu, 2008; Jiang et al., 2013), which prompted us to investigate whether sex hormones inhibit TMEM16A activity. Therefore, we recorded mouse and human TMEM16A currents induced by intracellular free calcium concentration (100 and 300 nM in mouse and human, respectively) during estrogen administration (Figure 4). In these experiments, four estrogens were applied at 100 μM, including estrone (E1), 17β-estradiol (E2), estriol (E3), and estetrol (E4). The mTMEM16A currents induced by intracellular free calcium concentration (100 nM) were inhibited by three estrogens, E2, E3, and E4 (Figures 4B–E). The % inhibition of E3 compared with Ani9 (10 µM) was significantly greater than that of E1 (Kruskal-wallis ANOVA, n = 5–6, Figure 4F). In mTMEM16A, the average % inhibition values were 5.04, 24.22, 56.79, and 22.88% for E1, E2, E3, and E4, respectively. In hTMEM16A, the average % inhibition values were 5.88%, 8.07%, 43.52%, and 16.43% for E1, E2, E3, and E4, respectively (Kruskal-wallis ANOVA, n = 5, Figure 4H–L). The inhibitory effect of E3 (100 μM) was potent in both mouse and human TMEM16A, and the inhibitory effects were reversible (Supplementary Figure S6). However, lower concentrations (1 and 10 µM) did not maintain similar levels of inhibition (Figures 4G,M). Because 100 µM of estrogen is drastically higher than physiological serum levels, these results suggest that the estrogen-induced TMEM16A inhibition is not significant in *vivo* situations.

### The Effect of Liquiritigenin on TMEM16A Depends on the Positional Relationship of the Hydroxyl Groups

To clarify the specificity of the chemical structure having inhibitory effect on TMEM16A, we focused on the positional relationship of hydroxyl groups in liquiritigenin because the hydroxyl groups at both ends of estrogen are reportedly important for binding to the estrogen receptor (Abot et al., 2014). Thus, we investigated the inhibitory effect of dihydrodaidzein, which is an analog of liquiritigenin that has a hydroxyphenyl at different carbon atom of pyranose (Spplementary Figure S7A). Dihydrodaidzein (100 μM) slightly inhibited mTMEM16A and hTMEM16A currents induced by intracellular free calcium at 100 and 300 nM, respectively (Supplementary Figure S7B,D). In mTMEM16A, the IC_50_ was 177.80 μM at a membrane potential of +60 mV (Supplementary Figure S7C). In hTMEM16A, the IC_50_ was 251.48 μM at a membrane potential of +60 mV (Supplementary Figure S7E). These results suggest that the inhibitory effect of dihydrodaidzein is weaker than that of liquiritigenin. Taken together, the positional relationship between the hydroxyl groups at both ends of a compound is important for TMEM16A inhibition.

## Discussion

The pharmacological effects of liquiritigenin in situations involving TMEM16A have been reported (Lee et al., 2014; Oh and Jung, 2016; Ramalingam et al., 2018; Crottes and Jan 2019). In this study, we found that liquiritigenin, which is a flavonoid derived from *Glycyrrhizae radix*, inhibited both mouse and human TMEM16A currents induced by intracellular free calcium. Moreover, liquiritigenin inhibited TMEM16A without agonistic effects on TRPV1 and TRPA1, which activate primary sensory neurons involved in pain sensation (Supplementary Figure S5). Therefore, the analgesic effect of liquiritigenin could be explained by this selective TMEM16A inhibition. In particular, it has been reported that liquiritigenin inhibited thermal pain sensation involving TRPM3 (Suzuki et al., 2016), and liquiritigenin reportedly inhibits the nonselective cation channel expressed in primary sensory neurons (Straub et al., 2013). Taken together, it suggests that the dual inhibition of TMEM16A and TRPM3 could be important to reduce pain sensation, similar to the relationship between TRPV4 and TRPA1 (Kanju et al., 2016).

To reduce pain sensation, the oral administration is convenient method. However, the intake of *Glycyrrhizae radix* itself could be not significant because the maximum plasma concentration of liquiritigenin after a single oral administration of 1 g/kg *Glycyrrhizae radix* extract is approximately 40 nM (Han et al., 2019), which is much lower than the IC_50_ for TMEM16A (Figures 1, 3). Although it is necessary to investigate the administration method, the oral administration of liquiritigenin or its precursor, liquiritin, could be more promising natural compound. Furthermore, liquiritigenin did not affect TMEM16B (Figure 2). TMEM16B is reportedly involved in anxiety-related behavior (Li et al., 2019). TMEM16B is expressed in GABAergic neurons of the amygdala, and TMEM16B-deficient mice are hyperactive in situations in which normal mice show anxiety. Because the inhibitory effect of liquiritigenin on TMEM16B currents was quite small (Figure 2), these abnormal behaviors might not be triggered by liquiritigenin administration itself. Meanwhile, there is possibility that the strong inhibition of TMEM16A by systemic administration potentially have severe side effects. TMEM16A deficiency induces tracheomalacia and subsequently causes postnatal lethality (Rock et al., 2008). Additionally, it has been reported that TMEM16A activation promotes insulin release from pancreatic β cells (Xu et al., 2014; Crutzen et al., 2016). Namely, the local administration of liquiritigenin may be advantageous.

In addition, a recent study indicated that TMEM16A is involved in virus infections (Pearson et al., 2020). This report showed that TMEM16A inhibitors suppress human respiratory syncytial virus. Moreover, the influenza A virus could also be suppressed by TMEM16A inhibitors. Furthermore, an anti-influenza effect of liquiritigenin has been reported (Grienke et al., 2014). These previous reports and our findings indicate the possibility that liquiritigenin-induced TMEM16A inhibition protects against virus infection in the apparatus respiratorius.

We also demonstrated estrogen-dependent inhibition of TMEM16A. Endogenous inhibition of TMEM16A has not been reported to our knowledge, although TMEM16A suppression by intracellular protons has been described (Chun et al., 2015). Among estrogen, E3 had the strongest effect on TMEM16A (Figure 4). E3 is mainly synthesized in the placenta from 16α-hydroxydehydroepiandrosterone, which is generated in the fetal liver (Thomas and Potter, 2013; Durkovic et al., 2014). These facts indicate that TMEM16A activity is down-regulated during pregnancy. However, estrogen-induced TMEM16A inhibition was not observed in physiological concentration ranges, although the serum concentration of E3 itself is unclear. Thus, the inhibitory effect of estrogen on TMEM16A is probably not physiologically relevant.

When estrogen binds to estrogen receptor α, the diagonal hydroxyl groups in the chemical structure are important for the formation of hydrogen bonds between estrogen and the receptor (Abot et al., 2014). This structural property may also be important for the binding between liquiritigenin and TMEM16A. We showed that dihydrodaidzein exerted a weaker inhibitory effect on TMEM16A currents than that of liquiritigenin (Supplementary Figure S7). Intriguingly, the inhibition of TMEM16A currents by daidzein (100 µM), which is an analog of dihydrodaidzein, is also clearly weaker than that by liquiritigenin, according to a previous report (Zhang et al., 2017). Therefore, these results suggest the possibility that the hydroxyl group positions at both ends of liquiritigenin are important structural features for TMEM16A inhibition.

Taken together, our novel findings could connect the fragmented knowledge that currently exists regarding physiological and pathological mechanisms involving TMEM16A and liquiritigenin.

## Data Availability

The raw data supporting the conclusions of this article will be made available by the authors, without undue reservation.
