# Supplementary material for: The Calcium-Activated Chloride Channel TMEM16A is Inhibitied by Liquiritigenin
Source: Front Pharmacol. 2021 Apr 8;12:628968. doi: 10.3389/fphar.2021.628968 (PMC8060913; doi:10.3389/fphar.2021.628968)
Supplement: Supplementary file 1 [file datasheet1.pdf]

Supplementary Material

1.1 Supplementary Figures

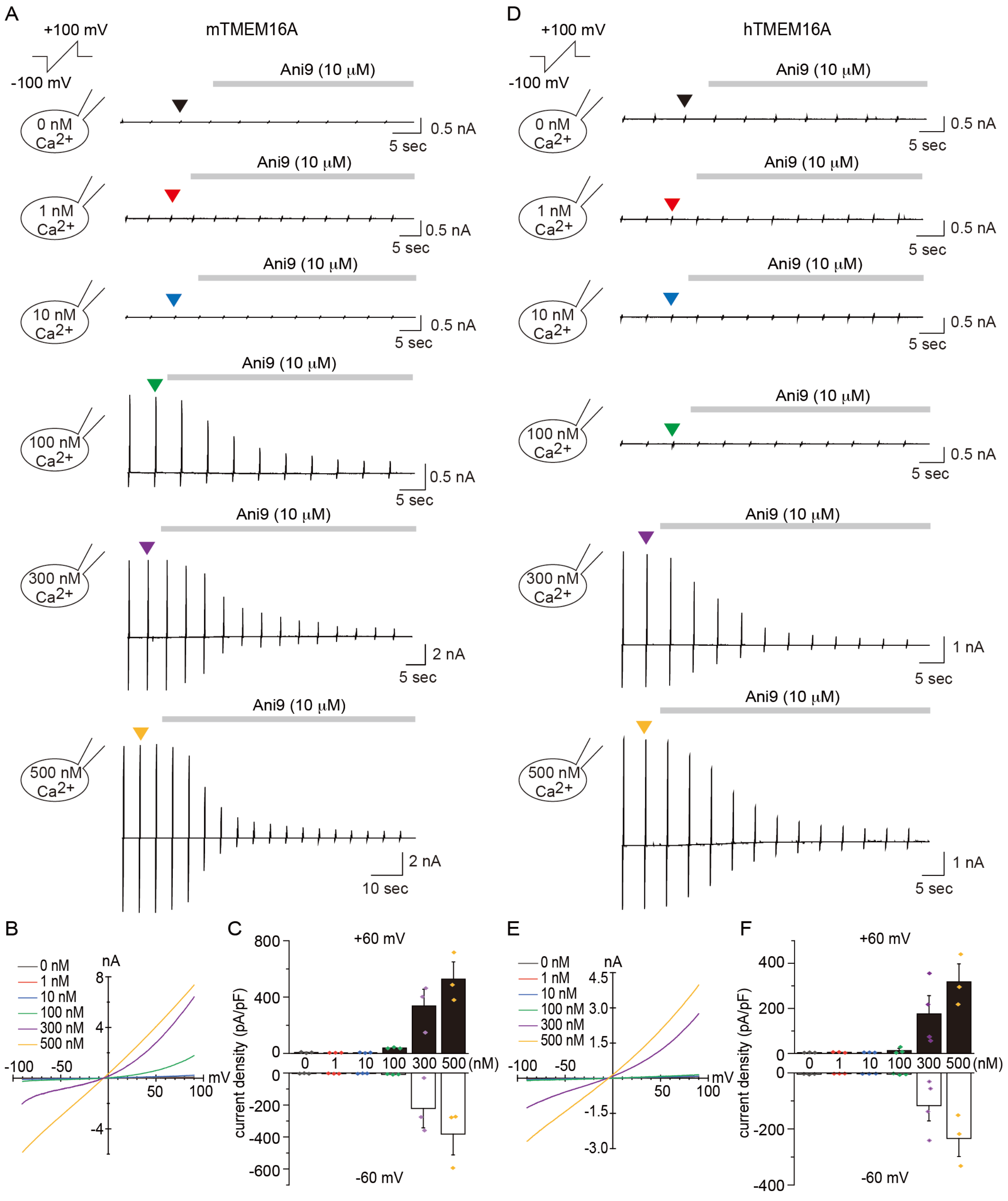

Supplementary Figure 1. Calcium-dependent TMEM16A activation. **(A–B)** Typical traces **(A)** and current-voltage curves **(B)** of chloride currents induced by 0, 1, 10, 100, 300, and 500 nM intracellular free calcium in HEK293T cells expressing mTMEM16A. Current-voltage curves at the time and different free calcium concentrations indicated by black (0 nM), red (1 nM), blue (10 nM), green (100 nM), violet (300 nM), and yellow (500 nM) arrowheads in A. **(C)** Mean values of current densities at −60 and +60 mV membrane potentials in HEK293T cells expressing mTMEM16A (n = 3 cells). **(D–E)** Typical traces **(D)** and current-voltage curves **(E)** of chloride currents induced by 0, 1, 10, 100, 300, and 500 nM intracellular free calcium in HEK293T cells expressing hTMEM16A. Current-voltage curves at the time and different free calcium concentrations indicated by black (0 nM), red (1 nM), blue (10 nM), green (100 nM), violet (300 nM), and yellow (500 nM) arrowheads in D. **(F)** Mean values of current densities at −60 and +60 mV membrane potentials in HEK293T cells expressing hTMEM16A (n = 3−4 cells). The holding potential was 0 mV, and ramp pulses (−100 to +100 mV, 300 ms) were applied every 5 s.

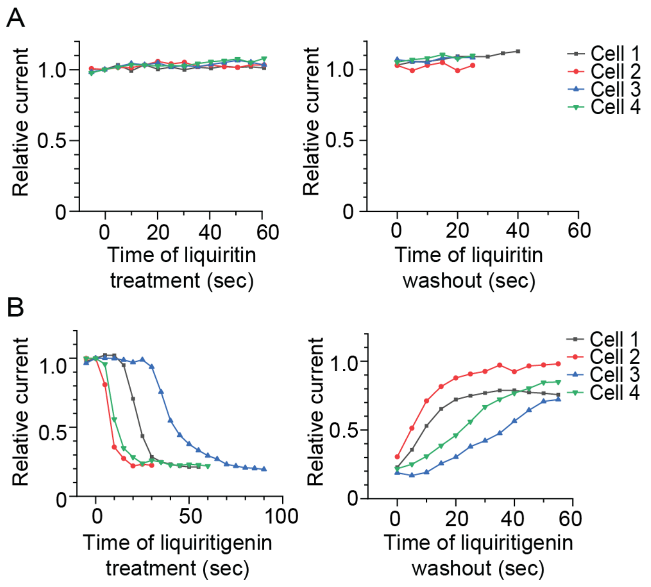

Supplementary Figure 2. The reversibility of the effect of liquiritigenin on mTMEM16A. **(A–B)** Time course of changes in relative mTMEM16A currents induced by 100 nM intracellular free calcium at +60 mV during treatments (left) of 100 μM liquiritin **(A)** and 100 μM liquiritigenin **(B)** and washout (right). The holding potential was 0 mV, and ramp pulses (−100 to +100 mV, 300 ms) were applied every 5 s.

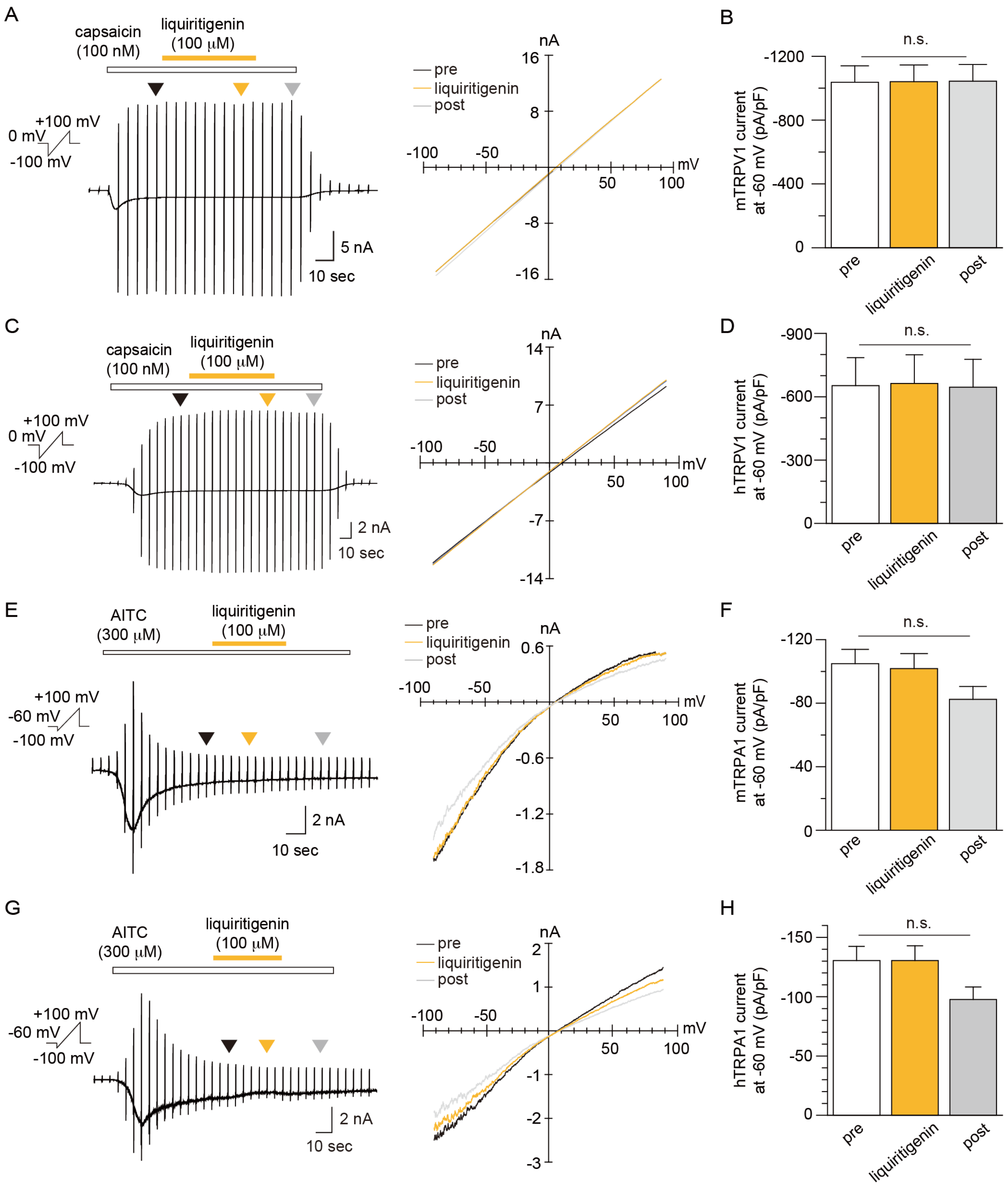
A
capsaicin
(100 nM)
liquiritigenin
(100 μM)
+100 mV
0 mV
-100 mV
5 nA
10 sec
nA
pre
liquiritigenin
post
16
8
-8
-16
-100
-50
50
100
mV
B
n.s.
-1200
-800
-400
0
mTRPV1 current
at -60 mV (pA/pF)
pre
liquiritigenin
post
C
capsaicin
(100 nM)
liquiritigenin
(100 μM)
+100 mV
0 mV
-100 mV
2 nA
10 sec
nA
14
7
-7
-14
D
n.s.
-900
-600
-300
0
hTRPV1 current
at -60 mV (pA/pF)
E
AITC
(300 μM)
liquiritigenin
(100 μM)
+100 mV
-60 mV
-100 mV
2 nA
10 sec
nA
0.6
-0.6
-1.2
-1.8
F
n.s.
-120
-80
-40
0
mTRPA1 current
at -60 mV (pA/pF)
G
AITC
(300 μM)
liquiritigenin
(100 μM)
+100 mV
-60 mV
-100 mV
2 nA
10 sec
nA
2
1
-1
-2
-3
H
n.s.
-150
-100
-50
0
hTRPA1 current
at -60 mV (pA/pF)

Supplementary Figure 3. Liquiritigenin did not exhibit inhibitory effects on TRPV1 and TRPA1. **(A–D)** Typical traces, current-voltage curves, and amplitudes of capsaicin (100 nM)-activated mTRPV1 **(A, B)** and hTRPV1 **(C, D)** currents during the application of 100 μM liquiritigenin to HEK293T cells (n = 10 cells each). The holding potential was 0 mV, and ramp pulses (−100 to +100 mV, 300 ms) were applied every 5 s. **(E–H)** Typical traces, current-voltage curves, and amplitudes of allyl isothiocyanate (AITC, 300 μM)-activated mTRPA1 **(E, F)** and hTRPV1 **(G, H)** currents during the application of 100 μM liquiritigenin to HEK293T cells (n = 10 cells each). The holding potential was −60 mV, and ramp pulses (−100 to +100 mV, 300 ms) were applied every 5 s. Current-voltage curves at the time indicated by black (pre-treatment), yellow (liquiritigenin), and gray (post-treatment) arrowheads in typical traces. Data are shown as the mean ± SEM; n.s., not significant, Kruskal-wallis ANOVA.

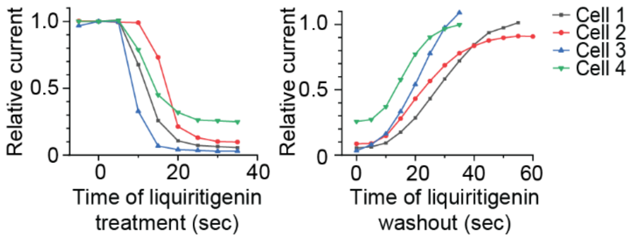

Supplementary Figure 4. The reversibility of the effect of liquiritigenin on hTMEM16A. Time course of changes in relative hTMEM16A currents induced by 300 nM intracellular free calcium at +60 mV during treatment of 100 μM liquiritigenin (left) and washout (right). The holding potential was 0 mV, and ramp pulses (−100 to +100 mV, 300 ms) were applied every 5 s.

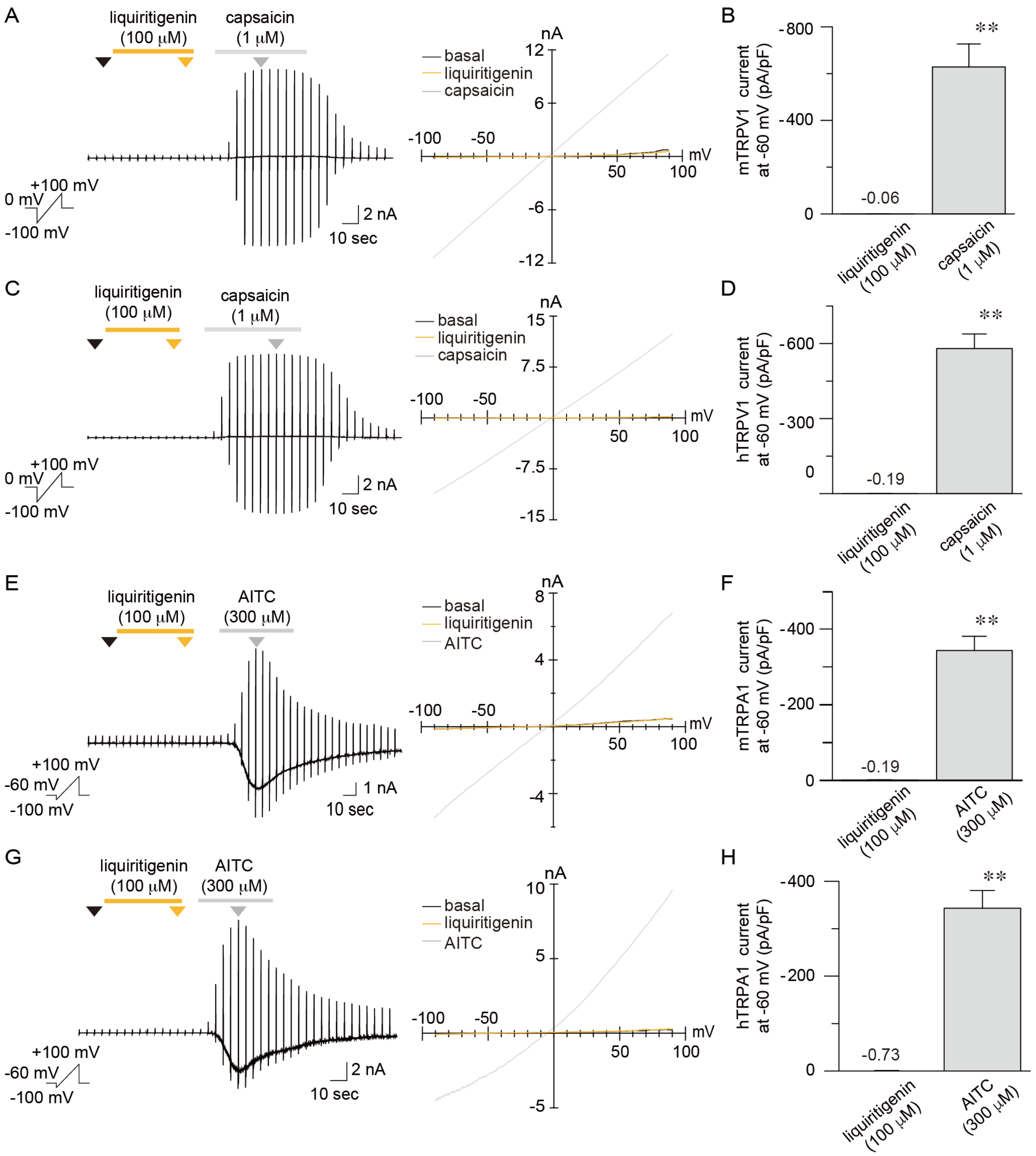

Supplementary Figure 5. Liquiritigenin exhibited no agonistic effects on TRPV1 and TRPA1. **(A–D)** Typical traces, current-voltage curves, and amplitudes of TRPV1 currents during the application of 100 µM liquiritigenin to HEK293T cells expressing mTRPV1 **(A, B)** and cells expressing hTRPV1 **(C, D)** (n = 10 cells each). The holding potential was 0 mV, and ramp pulses (−100 to +100 mV, 300 ms) were applied every 5 s. Typical current-voltage curves at the time indicated by black (basal), yellow (liquiritigenin), and gray (capsaicin) arrowheads. **(E–H)** Typical traces and amplitudes of TRPA1 currents during the application of 100 µM liquiritigenin to HEK293T cells expressing mTRPA1 **(E, F)** and hTRPA1 **(G, H)** (n = 10 cells each). The holding potential was −60 mV, and ramp pulses (−100 to +100 mV, 300 ms) were applied every 5 s. Typical current-voltage curves at the time indicated by black (basal), yellow (liquiritigenin), and gray (AITC) arrowheads. Data are shown as the mean ± SEM; $^{**}P < 0.01$, Wilcoxon matched-pairs signed rank test.

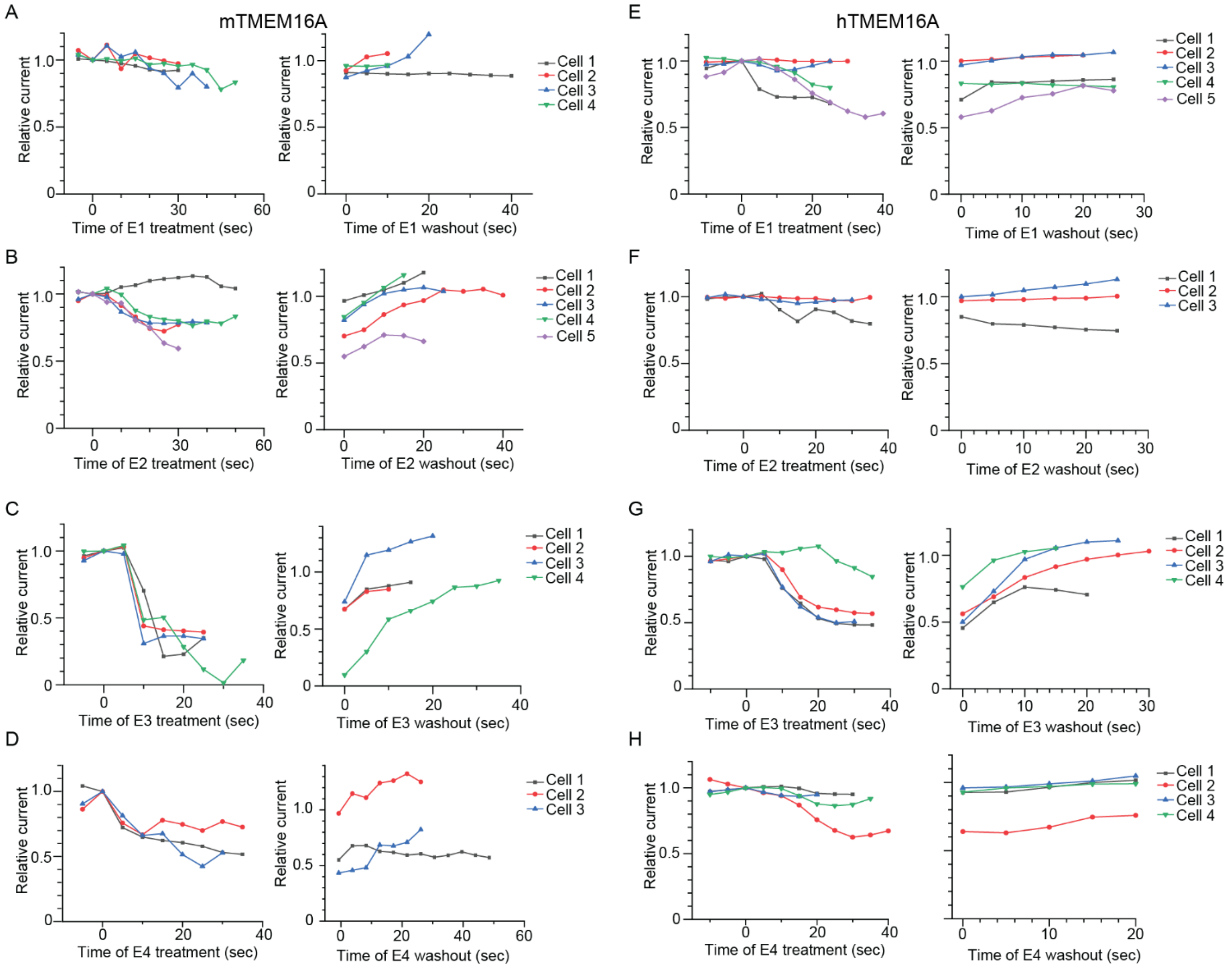

Supplementary Figure 6. The reversibility of the effects of estrogen on mTMEM16A and hTMEM16A. **(A–D)** Time course of changes in relative mTMEM16A currents induced by 100 nM intracellular free calcium at +60 mV during treatments (left) and washout (right) of 100 µM E1 **(A)**, E2 **(B)**, E3 **(C)**, and E4 **(D)**. The holding potential was 0 mV, and ramp pulses (−100 to +100 mV, 300 ms) were applied every 5 s. **(E–H)** Time course of changes in relative hTMEM16A currents induced by 300 nM intracellular free calcium at +60 mV during treatments (left) and washout (right) of 100 µM E1 **(E)**, E2 **(F)**, E3 **(G)**, and E4 **(H)**. The holding potential was 0 mV, and ramp pulses (−100 to +100 mV, 300 ms) were applied every 5 s.

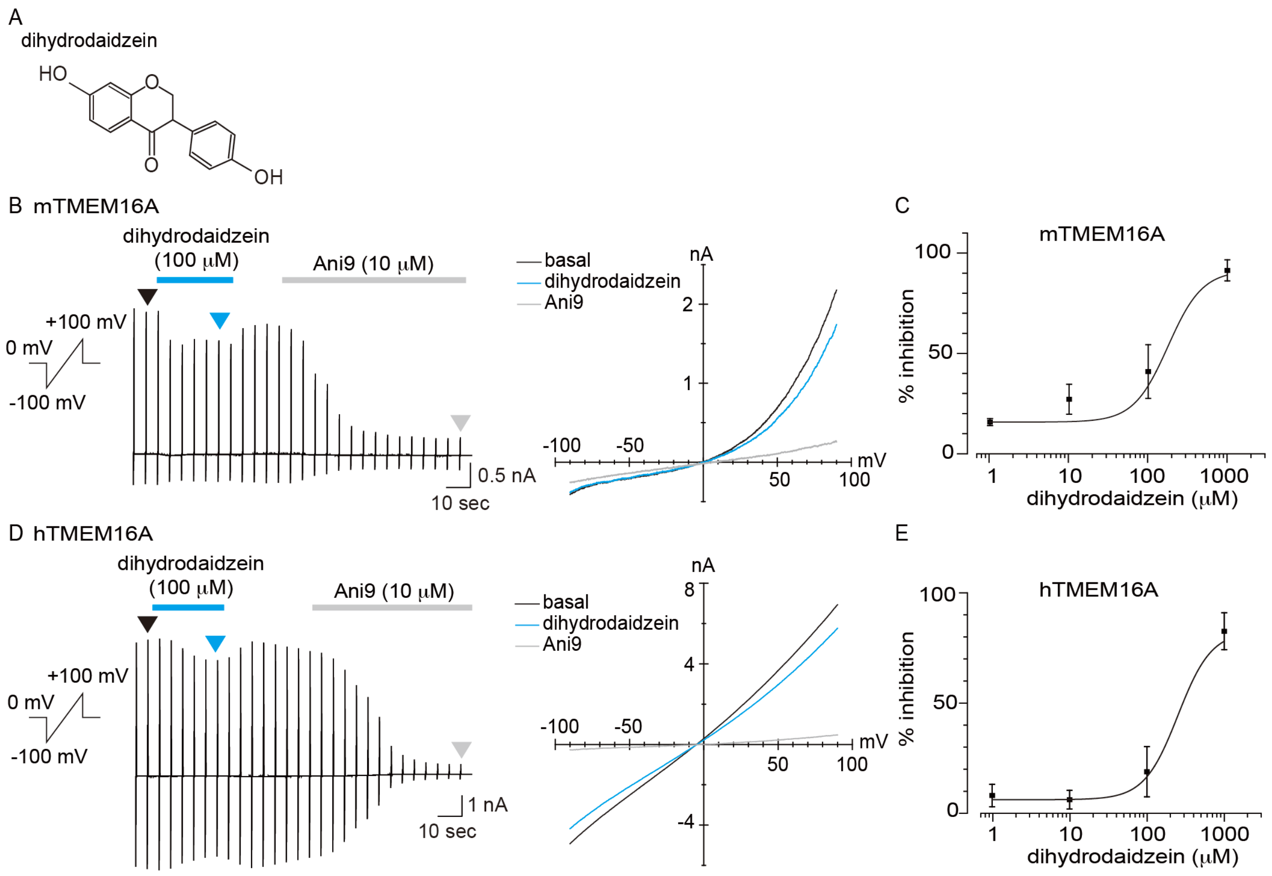

Supplementary Figure 7. The inhibitory effects of dihydrodaidzein on mTMEM16A and hTMEM16A. **(A)** Chemical structure of dihydrodaidzein. **(B)** Typical trace (left) and current-voltage curves (right) of chloride currents in HEK293T cells expressing mTMEM16A. Basal TMEM16A currents were induced by 100 nM intracellular free calcium. Current-voltage curves at the time indicated by black (basal), cyan (dihydrodaidzein), and gray (Ani9) arrowheads (left). **(C)** Dose-response curve for dihydrodaidzein-induced inhibition of mTMEM16A currents at +60 mV (n = 3−4 cells). The IC_{50} was 177.80 μM. **(D)** Typical trace (left) and current-voltage curve (right) of chloride currents in HEK293T cells expressing hTMEM16A. Basal hTMEM16A currents were induced by 300 nM intracellular free calcium. Current-voltage curves at the time indicated by black (basal), cyan (dihydrodaidzein), and gray (Ani9) arrowheads (left). **(E)** Dose-response curve for dihydrodaidzein-induced inhibition of hTMEM16A currents at +60 mV (n = 3 cells). The IC_{50} was 251.48 μM. The holding potential was 0 mV, and ramp pulses (−100 to +100 mV, 300 ms) were applied every 5 s.
